# Online Information Exchange and Anxiety Spread in the Early Stage of the Novel Coronavirus (COVID-19) Outbreak in South Korea: Structural Topic Model and Network Analysis

**DOI:** 10.2196/19455

**Published:** 2020-06-02

**Authors:** Wonkwang Jo, Jaeho Lee, Junli Park, Yeol Kim

**Affiliations:** 1 The Institute for Social Data Science Pohang University of Science and Technology Pohang Republic of Korea; 2 National Cancer Control Institute National Cancer Center Goyang Republic of Korea; 3 Department of Family Medicine National Cancer Center Goyang Republic of Korea

**Keywords:** coronavirus, anxiety, pandemic, online, health information exchange, topic modeling

## Abstract

**Background:**

In case of a population-wide infectious disease outbreak, such as the novel coronavirus disease (COVID-19), people’s online activities could significantly affect public concerns and health behaviors due to difficulty in accessing credible information from reliable sources, which in turn causes people to seek necessary information on the web. Therefore, measuring and analyzing online health communication and public sentiment is essential for establishing effective and efficient disease control policies, especially in the early stage of an outbreak.

**Objective:**

This study aimed to investigate the trends of online health communication, analyze the focus of people’s anxiety in the early stages of COVID-19, and evaluate the appropriateness of online information.

**Methods:**

We collected 13,148 questions and 29,040 answers related to COVID-19 from Naver, the most popular Korean web portal (January 20, 2020, to March 2, 2020). Three main methods were used in this study: (1) the structural topic model was used to examine the topics in the online questions; (2) word network analysis was conducted to analyze the focus of people’s anxiety and worry in the questions; and (3) two medical doctors assessed the appropriateness of the answers to the questions, which were primarily related to people’s anxiety.

**Results:**

A total of 50 topics and 6 cohesive topic communities were identified from the questions. Among them, topic community 4 (suspecting COVID-19 infection after developing a particular symptom) accounted for the largest portion of the questions. As the number of confirmed patients increased, the proportion of topics belonging to topic community 4 also increased. Additionally, the prolonged situation led to a slight increase in the proportion of topics related to job issues. People’s anxieties and worries were closely related with physical symptoms and self-protection methods. Although relatively appropriate to suspect physical symptoms, a high proportion of answers related to self-protection methods were assessed as misinformation or advertisements.

**Conclusions:**

Search activity for online information regarding the COVID-19 outbreak has been active. Many of the online questions were related to people’s anxieties and worries. A considerable portion of corresponding answers had false information or were advertisements. The study results could contribute reference information to various countries that need to monitor public anxiety and provide appropriate information in the early stage of an infectious disease outbreak, including COVID-19. Our research also contributes to developing methods for measuring public opinion and sentiment in an epidemic situation based on natural language data on the internet.

## Introduction

The recent appearance of the novel coronavirus disease (COVID-19) has been devastating worldwide. In South Korea, hundreds of new cases have been diagnosed daily since late February 2020. The cumulative number of confirmed cases at the time of writing (April 1, 2020) exceeded 9000. Internationally, over 800,000 cases have been confirmed in more than 200 countries, areas, and territories [1], despite the World Health Organization’s request for global efforts to slow down the spread of the virus the previous month [2]. Most countries have strongly recommended basic preventive methods such as quarantine and isolation of suspected cases, a macrolevel campaign on improving personal hygiene (eg, more frequent hand washing), or using masks in public sites. Additionally, some countries including Korea are now implementing more severe measures such as a social distancing that require the general population to refrain from congregating in public places.

In the event of a population-wide infectious disease outbreak such as COVID-19 people’s online activities could significantly affect public concerns and health behaviors. Many studies have indicated people’s active use of online information in various crisis situations, including a public health crisis [3,4]. Information and emotional exchanges between people on the internet form public opinions and concerns, which in turn affects people’s cognition and behavior. Although these opinions and information on the internet are sometimes useful, they are not always appropriate. There could be dissemination of misinformation, which may lead to inappropriate medical advice or unnecessary anxiety [5-7].

Analyzing data on the internet that records how people voluntarily exchange opinions and information about COVID-19, such as relevant posts from social media services, provides a valuable opportunity to understand and monitor the public concerns over COVID-19 and the dissemination of related information on the internet. Considering the need to manage rumors and monitor public opinions and behaviors in the context of a mass infectious disease, and the importance of internet opinions in the event of a population-wide outbreak, the analysis of the online data has great implications for the formation of efficient and effective health policies and appropriate provision of information. These spontaneously written language materials contain a wealth of information about various topics on COVID-19 that cannot be thoroughly predicted by health policy makers and public health researchers, and thus cannot be measured by a traditional predetermined questionnaire. Hence, analyzing web-based data can supplement traditional surveys and contribute to making health policies for the general population [7-9].

Web data analysis is particularly valuable in the early stage of an infectious disease outbreak. In the early stage of a new disease outbreak, health authorities may lack proper guidelines for the disease, and people may not find trustworthy information from other sources. Because of this situation, people might be more affected by uncertain information on the internet. Thus, monitoring web data in the early stage of an infectious disease outbreak is important to prevent inappropriate dissemination of misinformation or unnecessary anxiety that could occur in the early stage of an outbreak.

This study primarily evaluated the public concerns over COVID-19 in the outbreak’s early stage using data from the online social questions and answers (Q&A) forum in Korea’s largest search engine, Naver.com [10,11], and analyzed the characteristics of item responses. The Naver Q&A forum (the service named “Jisik-In,” meaning “an intellectual” in Korean) resembles Quora.com, as it allows users to post questions and answers on any topic. We used 13,148 questions and threaded answers posted on Naver’s Q&A forum in the early stage of the COVID-19 outbreak to analyze the characteristics of the public’s online concerns and the appropriateness of information circulated there.

In summary, our research questions include the following:

1. What is the focus of the questions on COVID-19 in the Naver Q&A forum?
2. How do the subjects observed in numerous questions change by time and main events?
3. What are the main objects concerning anxiety and worry on COVID-19?
4. How appropriate or significant is the information provided in the answers to the questions communicating anxiety and worry?

## Methods

### Data Collection

This study used the question and answer data available on Naver’s Q&A forum. The forum is open to the public and allows individuals to post both questions and answers, anonymously or otherwise.

There were two main reasons for selecting Naver’s Q&A forum among numerous online services available for public opinion exchange. First, Naver is a service provider with a dominant market position in Korea. It receives about 30 million visitors daily and is estimated to be used by about 76% of Korean Internet users as the main search portal website based on a recent survey [10,12]. Moreover, Naver is the only search engine of massive users with a Q&A forum that allows users to freely access the information exchange. Although similar information exchanges could happen in other internet communities and social network services, in general, only their members could approach and see them, so their influence is limited. Therefore, Naver’s Q&A forum data better illustrates the Korean public’s concern generated through online postings. Second, Unlike data from other social media, language data from a Q&A forum includes a detailed context of the author’s interests and feelings. The question and answer form allows the user to post detailed information because its aim is to help others understand the full situation. A Twitter post, for example, often simply reveals an author’s feelings or anxieties; however, a question from Naver’s Q&A forum explains the issue’s background. Our data, thus, may allow more informative analysis on general public concerns over COVID-19 than other sources of web-based data.

COVID-19–related questions posted between January 20, 2020, and March 2, 2020, and their respective answers were collected from Naver’s Q&A forum and used for analysis. January 20, 2020, was chosen as the starting point for data collection since it corresponds with the diagnosis of the first patient with COVID-19 in Korea, and questions before this date are scarce. Duration of the previously mentioned data was then confirmed by considering the frequency and characteristics of the data during preprocessing. The procedure for identifying the COVID-19–related posts comprised several steps.

First, one question and its attached answers were considered as one document. We collected all the documents (questions and answers) containing the word “코로나” (in English, “corona”) from December 30, 2019, to March 2, 2020. This, however, also extracts questions and answers using the word “코로나” that refers to objects other than COVID-19.

Second, the data were reselected from the results of the first step using additional search criteria to select COVID-19–related questions and answers more accurately. The search criteria were as follows: ([코로나 or corona or 우한 or COVID] and [바이러스 or 폐렴]) or (코로나19 or 코로나 19 or 신종코로나 or 신종코로나 or COVID19 or COVID 19 or COVID-19). English and Korean words were mixed in the search criteria because Koreans use both languages commonly. The English translations of the Korean words used here are as follows: 코로나=corona, 우한=Wuhan, 바이러스=virus, 폐렴=pneumonia, 신종코로나=novel corona. Therefore, we included biological words such as virus or pneumonia, or a relatively formal name referring to this disease, such as COVID19 or corona19, in the search criteria. These criteria were used to identify questions and answers that were related to COVID-19 and contained at least a fraction of biological perspective, as we assumed that the biological words and formal name reflect the perspective at least a little. Since COVID-19 is a controversial issue in domestic politics, we searched for posts that contained at least some biological perspective and excluded posts written purely from a political viewpoint.

Third, we isolated the questions from the selected data and again selected the questions satisfying the following search criteria: (코로나 or corona or 우한 or COVID). Questions including irrelevant words (eg, pets, guitars) were removed. Since dogs or cats could be infected with a different type of coronavirus than COVID-19, there were questions about it. We excluded the questions related to pets. Questions related to “corona,” the guitar-producing company in Korea, were also excluded. The advanced criteria were applied only to the questions because some users supplied answers without considering the questions’ contents. In other words, although rare, there were cases in which answers related to COVID-19 were attached to questions unrelated to COVID-19. This ensured that both questions and answers were related to COVID-19.

Fourth, the duplicate questions were deleted. Q&A forum users sometimes reposted the same question or posted a similar question with a slight change in the wording, which were unhelpful. The criteria used to find duplicate questions were whether the first 50 or the last 50 characters of the question, including blank spaces, were duplicated.

**Figure 1:**
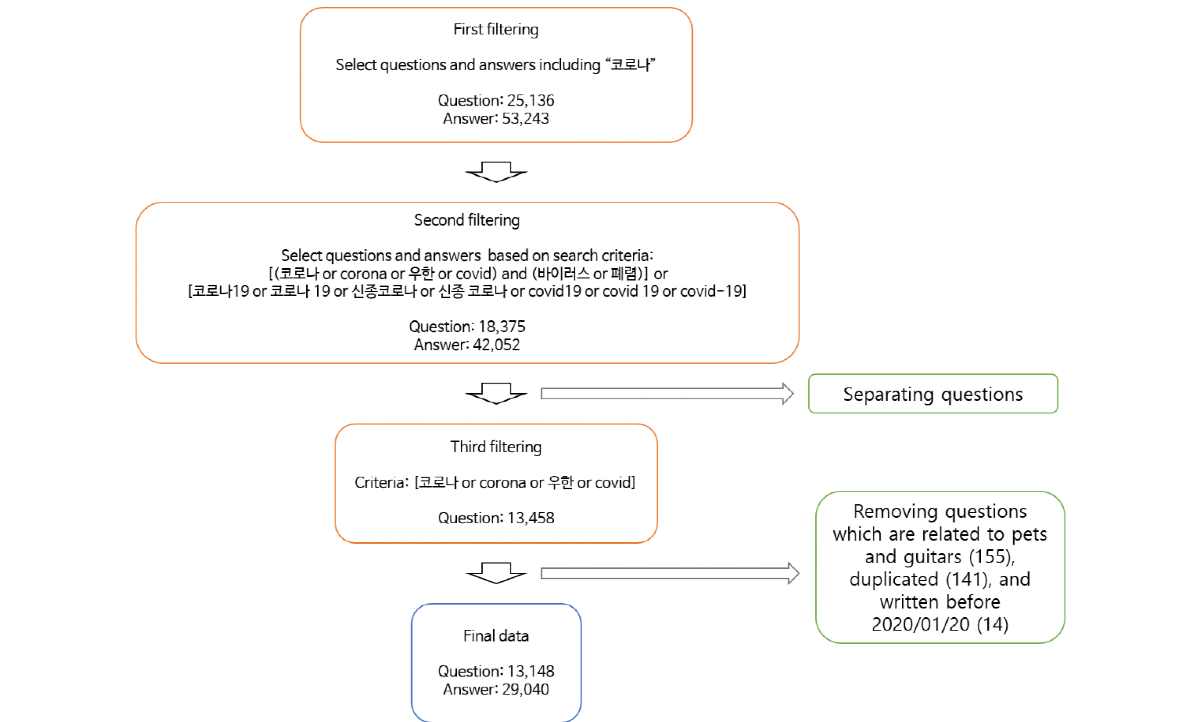
Data filtering process. COVID-19: coronavirus disease.

Fifth, the few questions (n=14) posted before January 20, 2020, were deleted, as the data in this period cannot represent public concern properly. As a result, 13,148 questions and 29,040 answers, which were assumed to be related to COVID-19, were collected. Figure 1 presents a schematic summary of this process.

### Data Analysis

Several text-mining techniques, including structural topic modeling and word network analysis, were used to analyze public concerns from 13,148 questions. Language data analyses have often used human interpretation capability [13]. The theme in language materials results from synthesizing various information, more than what is simply and explicitly expressed. Therefore, it is convenient to capture themes by mobilizing human ability to interpret texts. However, there is a clear limitation on the amount of data that can be processed by a few researchers. This explains why several previous studies analyzing medical-related media or internet posts used a small amount of sample data [14-16]. These studies were also susceptible to the unfavorable effects of human researchers’ subjectivity. Using text mining techniques to extract useful information from large volumes of data using computers, this study objectively estimated public concerns from large volumes of language data.

Although text mining allows us to examine a huge amount of data, these techniques usually cannot capture delicate nuances. For example, using basic text mining techniques, determining whether the answer is correct information or a rumor is somewhat difficult. This poses a challenge to the researcher because rumors, especially convincing rumors, use similar words and connection of words with valid information. To compensate for this, we also used a method that allows medical doctors (family medicine specialists) to categorize answers to questions on a particular topic and then analyze the characteristics of those answers.

In summary, three main methods were used for analysis: the structural topic model (STM), network analysis, and professional qualitative classification. Each method is described in the following sections.

#### Structural Topic Model

The STM, a type of topic modeling method, was selected to extract the overall themes or focus of 13,148 questions and examine how the theme or focus changed over time.

Like most topic modeling methods developed after the latent Dirichlet allocation (LDA), the STM can extract multiple topics and the topics’ probability distribution in each document from a large number of documents. The extracted topics and their distribution are information that summarizes the given documents [17,18].

The topic estimation process is based on several assumptions. Topic modeling methods assume that a document is a simple set of words and a topic is a probability distribution of words (eg, cat: 0.015, dog: 0.01, pet: 0.009, etc). Each document contains multiple topics with specific probability distributions (eg, the first document: topic 1=0.4, topic 2=0.2, and topic 3=0.4). It is then assumed that individual documents were randomly generated from the topics and their distribution per document, and were not directly written by humans; thus, the most probable topics and their distributions are estimated considering the given data [17-19].

Naturally, the probability distribution of words itself does not have intuitive meaning; however, we can interpret the meaning of the topic from the probability distribution of words. When a topic is expressed qualitatively, it mainly appears as an unequal use of words. For example, suppose a topic of “cancer screening test” exists in the language materials. Certain words like “screen” or “mammography” would be used more frequently in this material than other words. Therefore, if we can deduce the word probability distributions, which are likely to produce the given documents, we can also deduce the topics’ meanings by noting the high probability words in the corresponding probability distributions.

Distributions of topics in each document are also important information for interpreting the topics because they can be used to identify how each topic is realized into language material. We can also identify documents that have the highest proportion on each individual topic. For example, we can identify the top ten documents with the highest proportion of topic 2 among all documents. After reading the ten documents, we can understand the detailed context and intuitive meaning of topic 2 more accurately.

In short, the topic modeling method estimates topics and their probability distributions per document that explain the given documents appropriately. Although the probability distribution itself does not provide intuitive meaning, researchers could interpret the topics’ meaning.

Besides this general function of topic modeling, the STM estimates how much of the document’s meta information affects the topics’ proportion or content [20,21]. Meta information refers to other information that exists in the document outside the document’s content (eg, when a document was written or the type of author). The STM estimates how the meta information affects the proportion and content of the extracted topics. Given that this study primarily aimed to analyze how the topics of questions changed over time, these attributes of the STM were deemed appropriate to achieve our research objectives. This study estimated how the time of posting the question affected the proportion of question topics.

**Figure 2:**
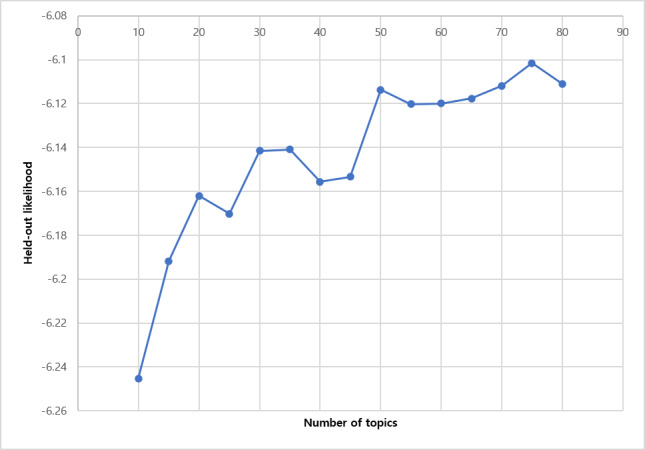
Held-out likelihood.

The application of the STM to the questions is described as follows. Of the 13,148 questions, 12 questions were additionally eliminated in the preprocessing for the STM. We only used the words that appeared in at least 2 questions and questions that contained at least 2 words, as a word that only appears in a single document or a question that contains only 1 word carries little information for topic modeling. We estimated 50 topics from the remaining 13,136 questions. The number of topics was set at 50 because there was no significant improvement in topic modeling performance after 50 as measured by the held-out likelihood [22] when changing the number of topics from 10 to 80 in increments of 5 (Figure 2). The posting time of the questions, which was measured by the unit of 1 day, was used as a covariate in our model for estimating topics’ proportion change by time.

All the topics were interpreted and labeled by considering three kinds of information: the words given high probability in each topic, the words with a high frequency and exclusivity (FREX) score in each topic, and the documents with a high proportion of each topic. The importance of the high probability words and the documents estimated to have a high proportion of each topic in interpreting topics has been explained previously. The words with a high FREX score supplement the high probability words by considering exclusivity and frequency together [21]. That is, a high FREX score word of a topic is important, especially in the topic. All the authors collectively interpreted and labeled 50 topics, considering the top 15 probability words, the top 15 FREX score words, and the top 10 questions with a high proportion of each topic.

We also used the STM to sample representative questions containing subjects related to anxiety and worry. To analyze the appropriateness of the answers to the questions related to the public’s anxiety and worry, most representative questions needed to be reselected from the entire batch of questions. Since it is impossible for a small group of human researchers to review 13,148 questions directly, the STM results were used to select questions that contained anxiety- and worry-related topics. We extracted questions that contained a high proportion of topics discussing physical symptoms and self-protection methods against COVID-19 because they were revealed as the main targets of anxiety and worry.

#### Network Analysis

Topic modeling is useful for identifying broad topics in a large volume of documents; however, a researcher cannot control the model to see results on specific subjects. Therefore, analyzing the relationship between particular words of interest is important in achieving our research goals.

One of our research objectives was to identify the objects and contexts of anxiety and worry expressed by people. To identify the source of anxiety and worry, we extracted the top 20 words that appeared most frequently in the questions including the words “불안” or “걱정,” which are Korean words for “anxiety” and “worry,” respectively. We considered both words appearing in the same question as a linkage or an association between the words.

Additionally, we created a word network using the top 50 words linked to the two Korean words referring to anxiety and worry to analyze the context of people’s anxiety and worry comprehensively. Observing individual words associated with anxiety and worry cannot accurately analyze the full context of anxiety and worry. A network of words linked to anxiety and worry allowed us to further analyze the context of anxiety and worry. Therefore, the top 50 words associated with the two Korean words referring to anxiety and worry were gathered, and the network was created. Connection criterion is a coappearance of words in the same question. This network is extremely dense because the network is made of words that are used in a similar context. Only the most prominent links were required to be selected to extract particularly prominent meanings. For this, only the links with the highest weight, (ie, 500 of the most frequent connections) were extracted to create a subnetwork. This subnetwork was assumed to contain the most prominent contexts related to people’s anxiety and worry.

The network community detection algorithm was applied to this subnetwork to extract distinguished themes from the network. This algorithm identifies relatively more cohesive communities of nodes within a network [23-25]. When applied to our word network, it worked to find a set of words that appeared more frequently with each other in the entire network. For example, the algorithm could judge that a “China,” “Wuhan,” “pneumonia,” “infection,” and “travel” word cluster appeared frequently in the entire word network. This was interpreted as a distinct theme that we found through network analysis. In other words, the network community detection algorithm identified the most prominent and distinct contexts that appeared when people expressed anxiety or worry. We identified cohesive communities of words, which contained at least 5 words because this amount was required to interpret the word community into a meaningful theme.

Walktrap was selected from the various network community detection algorithms because of its excellent performance while overcoming the “resolution problem” [25,26]. A resolution problem refers to a situation in which algorithms do not capture a community of a small number of nodes properly, a frequently faced problem in applying network community detection algorithms. Put simply, the Walktrap algorithm calculates the distance between nodes in the network using a random walk from each node and finds communities based on that distance. The analyst sets the step of the random walk for distance calculation, which we set to 2. The network analysis was implemented through the igraph package of R (R Foundation for Statistical Computing).

The Walktrap algorithm was also used to identify cohesive communities of topics related to each other. The STM estimates the correlation between topics [20,27]. A positive correlation between two topics means that the two topics are likely to appear together in the same document. We assumed this positive correlation to be a link between topics and formed a network of all topics. Next, using the network community detection algorithm, we analyzed whether there were relatively more cohesive communities of topics in the entire network of topics. In other words, we tried to identify sets of topics that were often expressed together. Consequently, we identified 6 topic communities that were judged to be cohesive. We labeled each topic community to express broader themes embracing topics belonging to the topic community, considering each topic’s interpretation made through the STM.

That is, after estimating 50 topics from 13,136 questions, these topics were summarized into 6 topic communities using the Walktrap algorithm and then interpreted. This additional step was employed due to the fact that, although 50 topics is a great summary, it is still a lot of information for a person to grasp intuitively.

#### Professionals’ Qualitative Coding

Using the results of the aforementioned methods, we assessed the appropriateness of the answers to questions dealing with the main targets of anxiety and worry, and found that the main themes were physical symptoms and self-protection methods. We selected sample questions and answers that addressed these two subjects. Two medical doctors, who are family medicine specialists and the authors of this paper, classified the answers into 5 independent categories: appropriate answers, unrelated answers, wrong answers, advertisement, and other. If there was a disagreement on an answer’s category, they discussed until they reached an agreement and recorded the agreed result.

The sample questions were selected by considering the proportion of topics in each question that resulted from using the STM. The themes of physical symptom and self-protection correlated with the fourth and fifth topic communities, respectively. Choosing high proportion questions from each community of topics could form good sample questions that appropriately represent each theme. Therefore, the proportion of the topic communities 4 and 5 was calculated for each question based on the topic proportion information per question. It was created by adding up the proportion of topics belonging to each topic community. The top 100 questions were selected for both topic communities 4 and 5, and the answers to each question were identified. The number of answers was 250 and 306, respectively.

#### Morphological Analysis and Part of Speech Tagging

For the previously mentioned methods, especially STM and network analysis, to function properly, morphemes need to be extracted from our language data. That is, we need information about what kinds of words appeared from where and at what frequency. The program used to determine this is called a morphological analyzer. From the many types of morphological analyzers applicable to Korean, Komoran was used because it is resilient to the spacing problem in Korean and is sufficiently qualified, as it won awards from the National Institute of Korean Language in 2016.

We would like to mention in advance a caveat regarding the words extracted from the data by Komoran; Korean and English do not respond 1:1. For example, there are several Korean words that can be translated as fever (“열” and “발열”). When translating such words into English, we numbered them (eg, fever_1, fever_2).

## Results

### Frequency of Documents and Words

**Figure 3:**
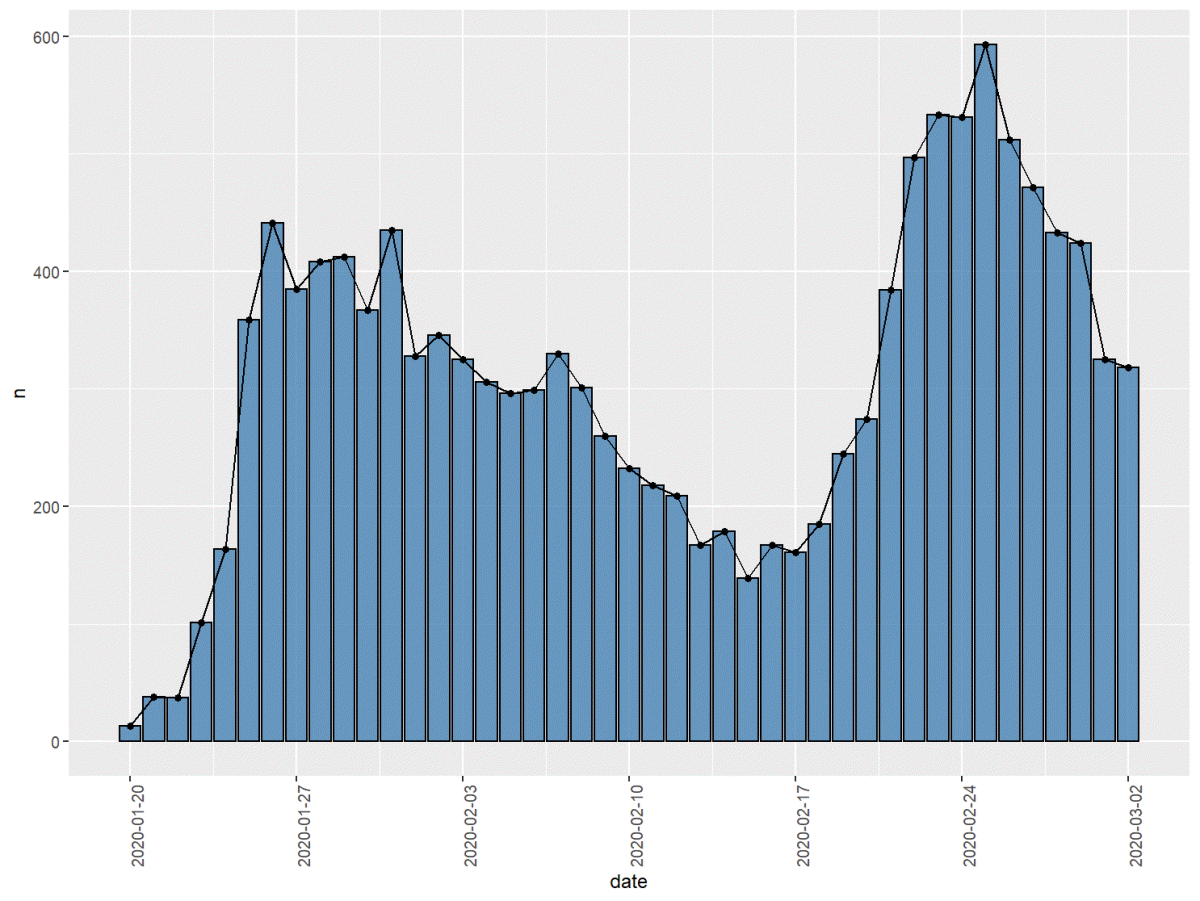
Number of questions by date.

Our data included a total of 13,148 questions. Figure 3 presents the number of questions sorted by date.

Of the words that appeared in the questions, Table 1 presents the top 30 frequency words. Those occupying the top 5 positions include “cough,” “symptom,” “throat,” “mask,” and “confirmed diagnosis.”

**Table 1:** Top 30 frequency words in all questions (nouns, excluding words containing “corona”).

Words	Frequency, n
Cough	5589
Symptom	4739
Throat	4177
Mask	2822
Confirmed diagnosis	2199
Human	2095
Cold	1983
Sputum	1953
Fever_1	1907
Degree	1887
Pneumonia	1844
House	1795
Worry	1792
Wuhan	1787
Hospital	1780
China	1691
Rhinorrhea	1432
Head	1427
Infection	1359
Work	1352
Recent	1278
Novel	1268
Headache	1246
Nose	1153
Feeling	1144
Test	973
Travel	958
Body	911
Anxiety	863
Virus	747

### Structural Topic Model

About 50 topics were estimated from 13,136 questions using the STM and were interpreted by the authors. Table 2 presents the results of the interpretation. The first column from the left is the topic number, the second column is the topic’s interpretation, and the third column is the topic community number that each topic belongs to. Topic numbers and topic community numbers are nominal numbers for distinction.

Most topics had an apparent subject or consistent contents that allowed a precise interpretation. Some topics, however, were extracted because of expressions that appeared repeatedly in various questions, regardless of the content of the question. This is because topic modeling captures the coappearance patterns of multiple words observed in documents without considering the meanings of words. For example, if many questions on different subjects contain similar expressions, like “please answer my question or you could be cursed,” the topic model would capture that pattern and estimate the topic based on the pattern. In our model, several topics were extracted because of unique Korean language usages. In this case, we unified the interpretation of the topic as “Questions involving particular Korean language expressions without a common subject.” Additionally, we presented the most prominent Korean expression in these topics in Korean in parentheses and translated its meaning into English.

**Figure 4:**
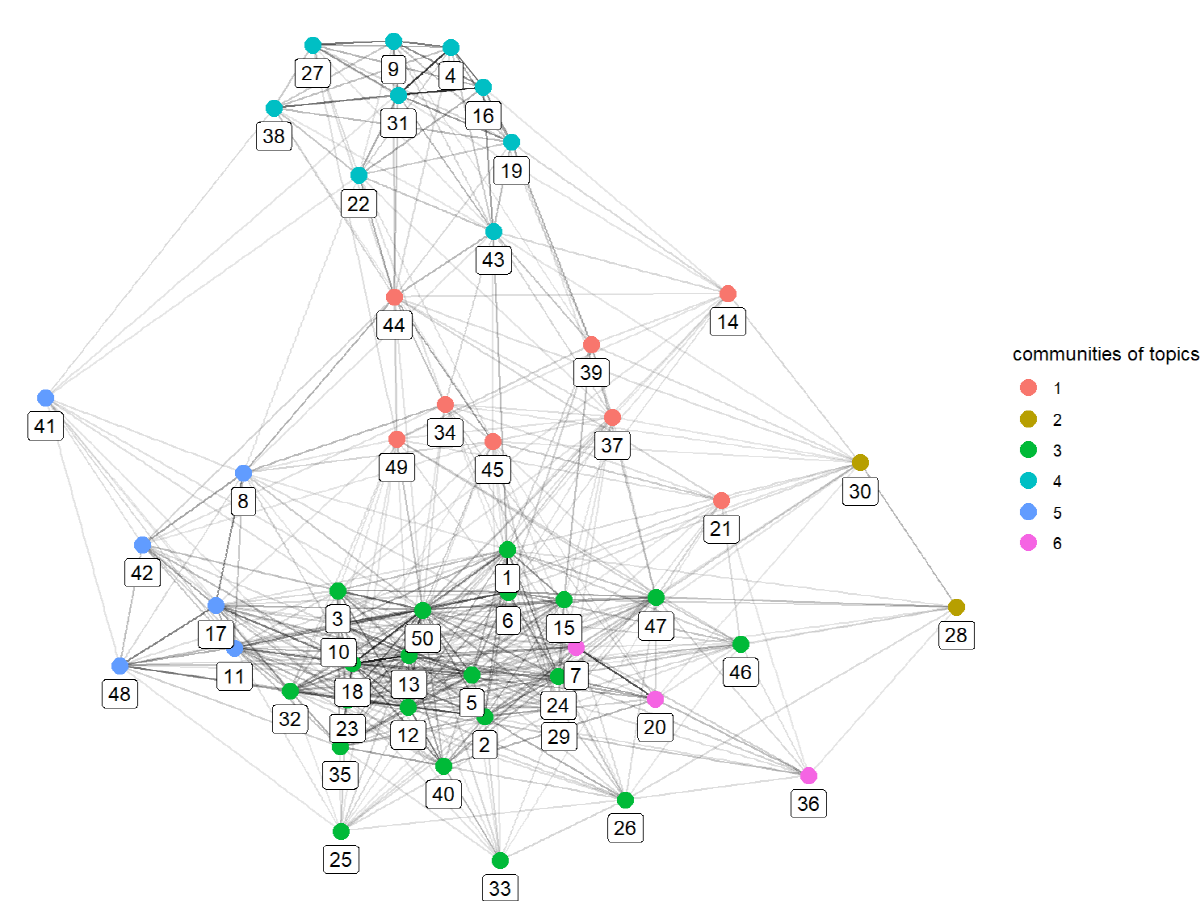
Topics network and topic communities.

The third column in Table 2 resulted from applying Walktrap to the topics’ correlation network for grouping topics into several cohesive communities. A total of 50 topics were categorized into 6 topic communities, each given a number (the rightmost column). The topics in Table 2 were sorted according to the topic community numbers to help readers easily identify topics belonging to each topic community. Figure 4 presents a visualization of the entire topics network and communities of topics identified through Walktrap. A node is a topic, the number below a node is the topic number, a link is a positive correlation between topics, and the color of the node indicates the topic grouping. Therefore, topics of the same color belong to the same community. Table 3 presents the result of the authors’ interpretations of topic communities.

**Figure 5:**
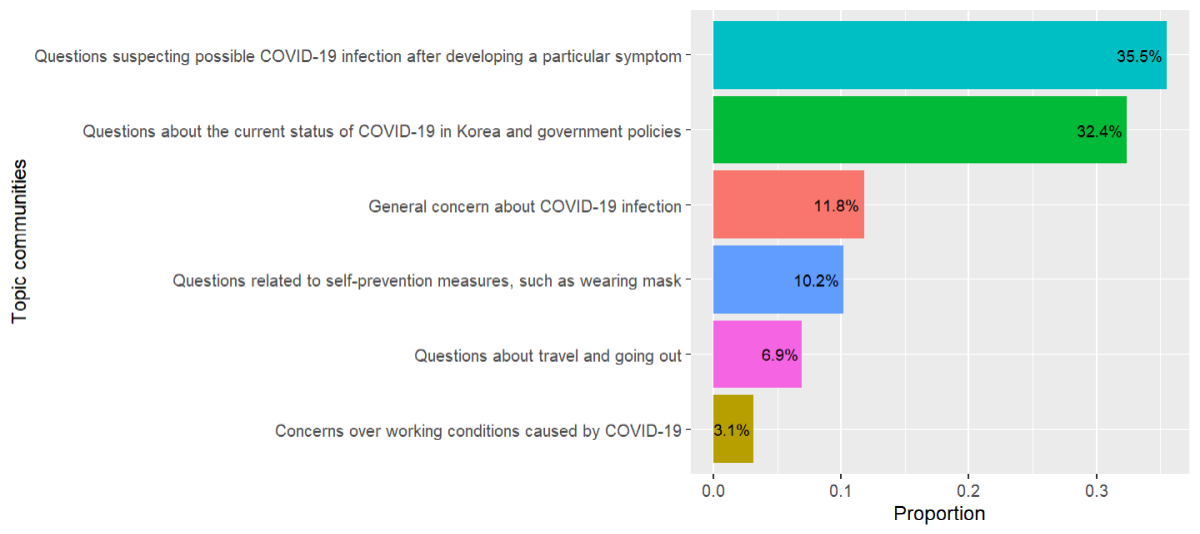
Proportion of topic communities in all questions. COVID-19: coronavirus disease.

**Figure 6:**
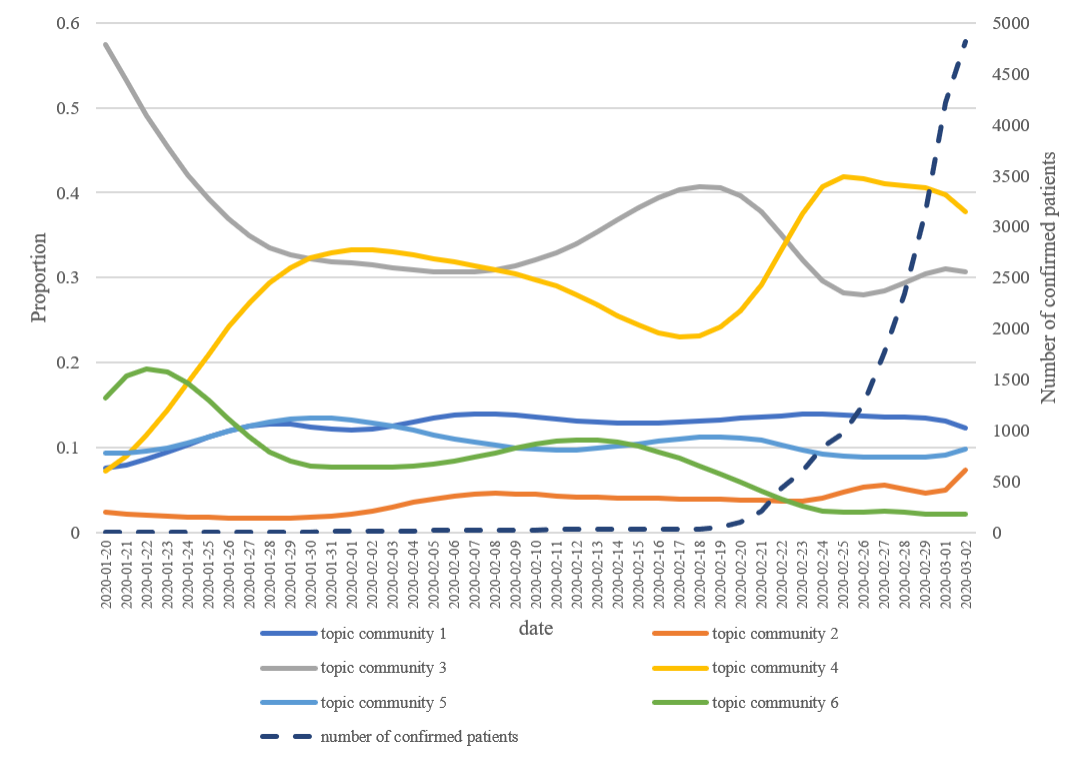
Proportion of topic communities by date.

The STM calculates the proportions of topics on a per-document basis. This allowed us to calculate the proportion of each topic in the entire document, which could also be used to calculate the proportion of the topic communities in the entire document. We aggregated proportions of topics belonging to the same community. Additionally, the STM calculated the proportion variation of all topics over time because the time variable was set as a covariate in our STM. The estimates of topics’ proportion changes over time could also be aggregated to produce the proportion change of the topic communities over time. Figures 5 and 6 present the results.

Topic community 4 (questions suspecting possible COVID-19 infection after developing a particular symptom) occupied the largest proportion of all the questions. It increased sharply in late February when the number of infections had begun to increase earnestly in Korea. In Figure 6, the yellow line (topic community 4) and the dashed line (the number of confirmed patients) are almost parallel after the second increase for topic community 4, the yellow line mimicking the increase in the number of confirmed patients. Moreover, it is noticeable that the portion of topic community 2 (concerns over working conditions caused by COVID-19) increased slightly as the COVID-19 situation became prolonged.

**Table 2:** Interpretations of topics.

Topic number	Interpretation	Topic community number
14	Questions on interpretation of English text related to COVID-19^a^	1
21	Questions on daily life that have little relevance to COVID-19	1
34	Questions on using public transport	1
37	Questions suspecting possible COVID-19 infection from past activity or experience	1
39	Questions on screening centers or hospital visits	1
44	Questions suspecting or worrying about possible COVID-19 infection by going outside or coming in contact with people	1
45	Questions suspecting or worrying about possible COVID-19 infection in their family	1
49	Questions involving particular Korean language expressions (“~때”: a noun indicating the particular point in time^b^; “~문제”: problem regarding ~) without a common subject	1
28	Questions regarding the impact of COVID-19 on their working conditions or days off work	2
30	Questions regarding part-time workers coping with the difficulties from COVID-19	2
1	Questions on the current status of the COVID-19 outbreak in Korea	3
2	Questions on the current status of the COVID-19 outbreak outside of Korea	3
3	Other questions regarding COVID-19	3
5	Questions on the expectation of further spread of the COVID-19 outbreak	3
6	Questions on the specific information of confirmed patients in a regional area, including patients’ “route map”	3
10	Questions regarding the end of the COVID-19 epidemic.	3
12	Questions involving particular Korean language expressions (“~가궁금하다”: I wonder~; “~를알려달라”: please explain about~) without a common subject	3
13	Questions expressing the author’s fear and difficulty related to COVID-19	3
15	Questions about treatment of COVID-19 in hospitals	3
18	Questions on specific circumstances that lead to COVID-19 infection	3
23	Questions involving particular Korean language expressions (“부탁드립니다”: could I ask~ please) without a common subject	3
24	Questions on the impact of the COVID-19 outbreak on the stock market	3
25	Questions relating to religion and donation	3
26	Questions about government policies on COVID-19, including an entry ban	3
29	Questions related to the Sincheonji Church and religious events	3
32	Questions involving particular Korean language expressions (“가능할까요”: is it possible to~) without a common subject	3
33	Questions involving particular Korean language expressions (“완전”: an adverb expressing the importance, such as completely or totally; “~겁니다”: an auxiliary verb indicating the current status^c^) without a common subject	3
35	Questions on a COVID-19 vaccine and treatment	3
40	Questions about Wuhan pneumonia in China	3
46	Questions on school opening and delay of school opening; questions on postponing the beginning of the school semesters and reopening date	3
47	Questions involving particular Korean language expressions (“~한데”: I feel like~ then; “~되는데”: it comes to~) without a common subject	3
50	Questions involving particular Korean language expressions (“요즘”: these days or at the moment) without a common subject	3
4	Questions suspecting possible COVID-19 infection after developing a sore throat	4
9	Questions suspecting possible COVID-19 infection after developing chest discomfort	4
16	Questions suspecting possible COVID-19 infection after developing a febrile sense and respiratory abnormality	4
19	Questions suspecting possible COVID-19 infection after developing a fever	4
22	Questions on the risk of catching COVID-19 from an exposure to cold weather or in day-to-day living	4
27	Questions suspecting possible COVID-19 infection after developing respiratory discomfort	4
31	Questions suspecting possible COVID-19 infection after developing a headache and cough	4
38	Questions suspecting possible COVID-19 infection after developing diarrhea and eating particular food	4
43	Questions suspecting possible COVID-19 infection after travelling	4
8	Questions about personal protection such as wearing a mask	5
11	Questions on the novel coronavirus contamination mechanism	5
17	Questions about how to use a mask	5
41	Questions on nonscientific knowledge related to COVID-19	5
42	Questions about hand sanitizer and parcels	5
48	Questions about disinfectants and air purifiers	5
7	Questions about the safety of traveling or visiting public places	6
20	Questions about safety related to travel	6
36	Questions on travel and flight cancellation procedures and fees	6

^a^COVID-19: coronavirus disease.

^b^Similar to *when* in a sentence (ie, *when* I was young).

^c^Similar to *have* in a sentence (ie, I *have* a headache).

**Table 3:** Interpretations of topic communities.

Topic community	Interpretation
1	General concern about COVID-19^a^ infection
2	Concerns over working conditions caused by COVID-19
3	Questions about the current status of COVID-19 in Korea and government policies
4	Questions suspecting possible COVID-19 infection after developing a particular symptom
5	Questions related to self-prevention measures such as wearing a mask
6	Questions about travel and going out

^a^COVID-19: coronavirus disease.

### Network Analysis

We extracted 20 words that were most frequently connected with words referring to anxiety and worry (in Korean, “불안” and “걱정,” respectively) from the questions posted from January 20 to March 1, 2020. Table 4 presents the results. In Table 4, the top 5 words include “cough,” “symptom,” “throat,” “mask,” and “cold.” This allowed us to infer that people’s anxiety was centered on physical symptoms and key self-protection methods, such as wearing masks.

**Figure 7:**
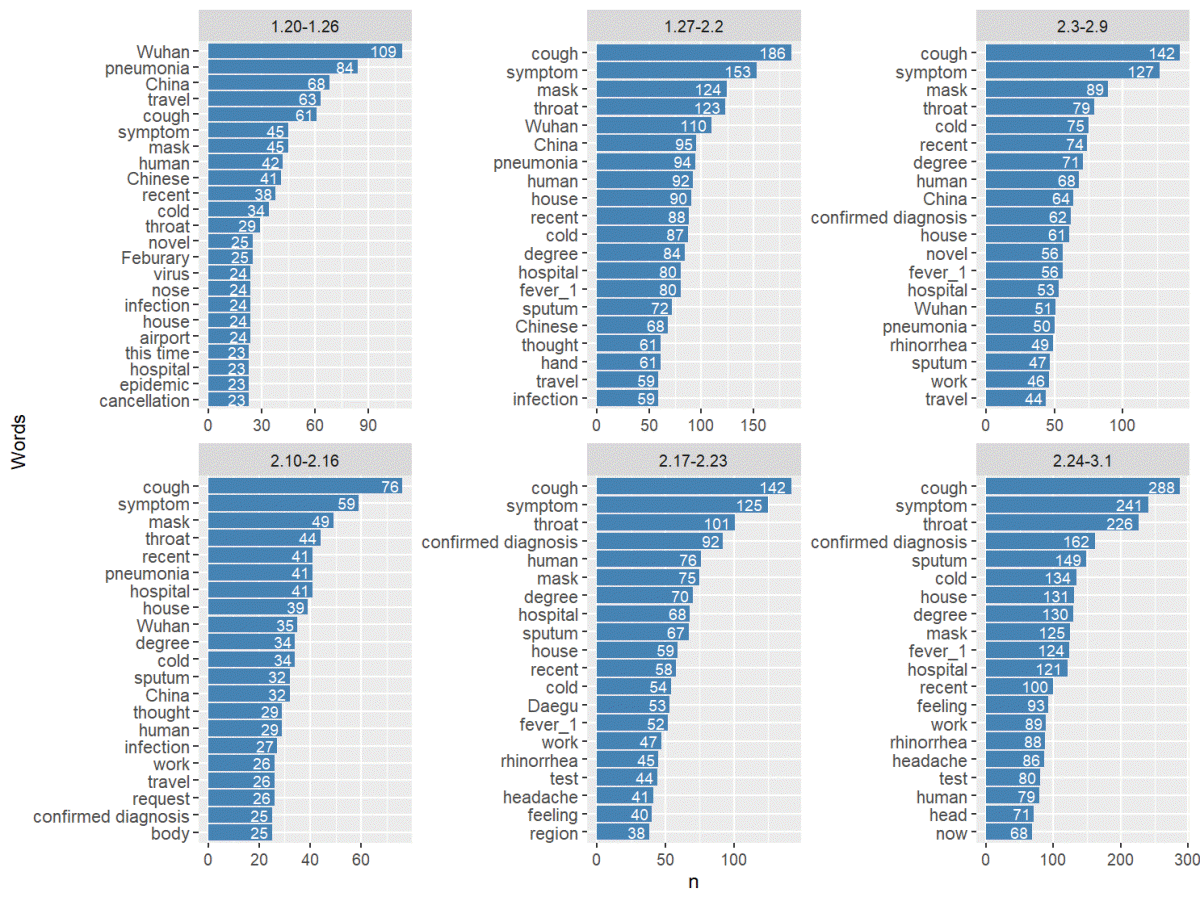
Top 20 frequency words appearing with anxiety and worry in all questions by week (nouns, excluding words containing “corona”).

We also checked whether the list of words associated with anxiety and worry would vary with time. The period (January 20-March 1, 2020) was divided into 6 weeks, and the top words associated with anxiety and worry was extracted from the data of the 6 subperiods (Figure 7). During the week of January 20-26, when the first confirmed COVID-19 cases were reported, the primary subject of anxiety was “China and traveling.” This was natural, considering that COVID-19 was limited to the Chinese Mainland at that time. However, as the number of confirmed COVID-19 cases increased in Korea, words related to physical symptoms appeared as the top words. The period after February 17 was when the number of confirmed cases increased sharply in South Korea by 2 or 3 digits. Henceforth, Koreans needed to be careful about contact with confirmed patients, and the word “confirmed diagnosis” emerged as the main word associated with anxiety. The word “mask” was consistently included in the top 10 words in all periods. In other words, the anxiety and worry about self-protection have been prevalent consistently throughout the entire period.

**Figure 8:**
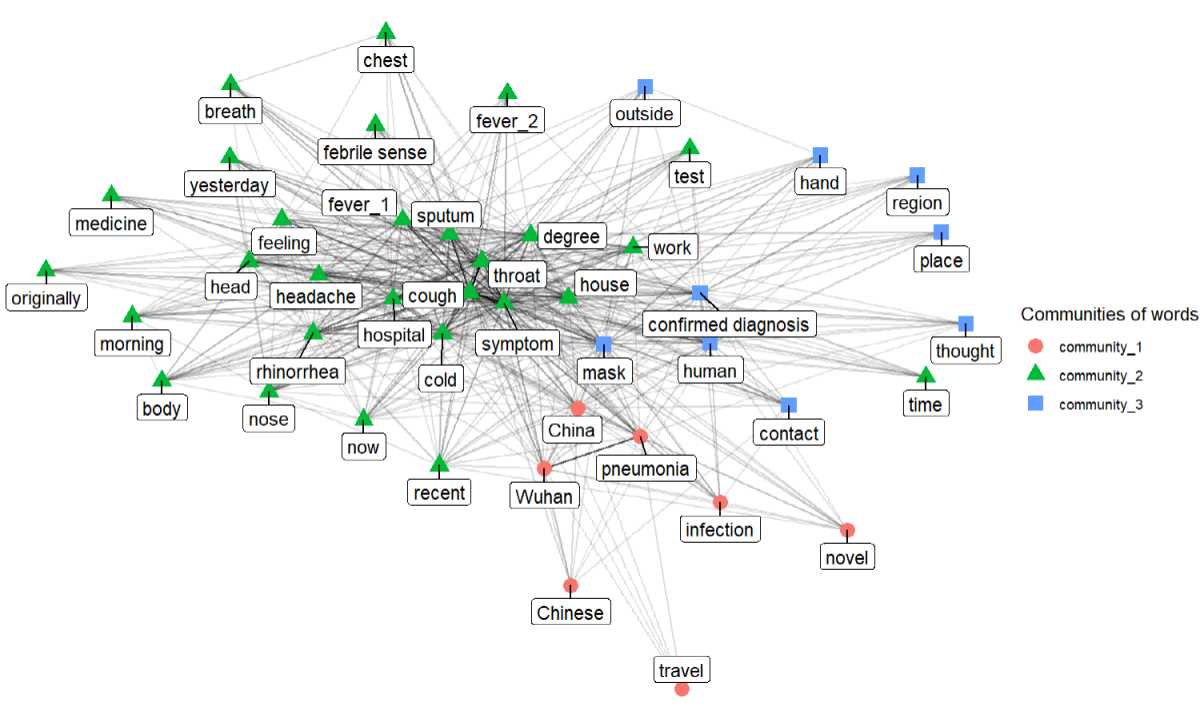
Word network of top 50 words linked to anxiety and worry.

We formed a word network using the top 50 words linked to anxiety and worry and extracted its subnetwork based on the most prominent 500 links. By applying the Walktrap algorithm to the subnetwork, we could extract three cohesive word communities or three distinct themes. Figure 8 presents a visualization of the three sets of words comprising at least 5 words. One is related to physical symptoms, another is related to self-protection, and the last is related to China. In other words, people’s anxiety was expressed in three main themes. This result was consistent with our reasoning based on the types of words linked to anxiety and worry.

**Table 4:** Top 20 frequency words appearing with anxiety and worry in all questions (nouns, excluding words containing “corona”).

Word	Frequency, n
Cough	895
Symptom	750
Throat	602
Mask	507
Cold	418
Degree	409
House	404
Confirmed diagnosis	400
Recent	399
Human	386
Hospital	386
Sputum	383
Fever_1	348
Wuhan	322
China	310
Pneumonia	308
Work	274
Rhinorrhea	271
Headache	245
Thought	238

### Professionals’ Qualitative Coding

**Figure 9:**
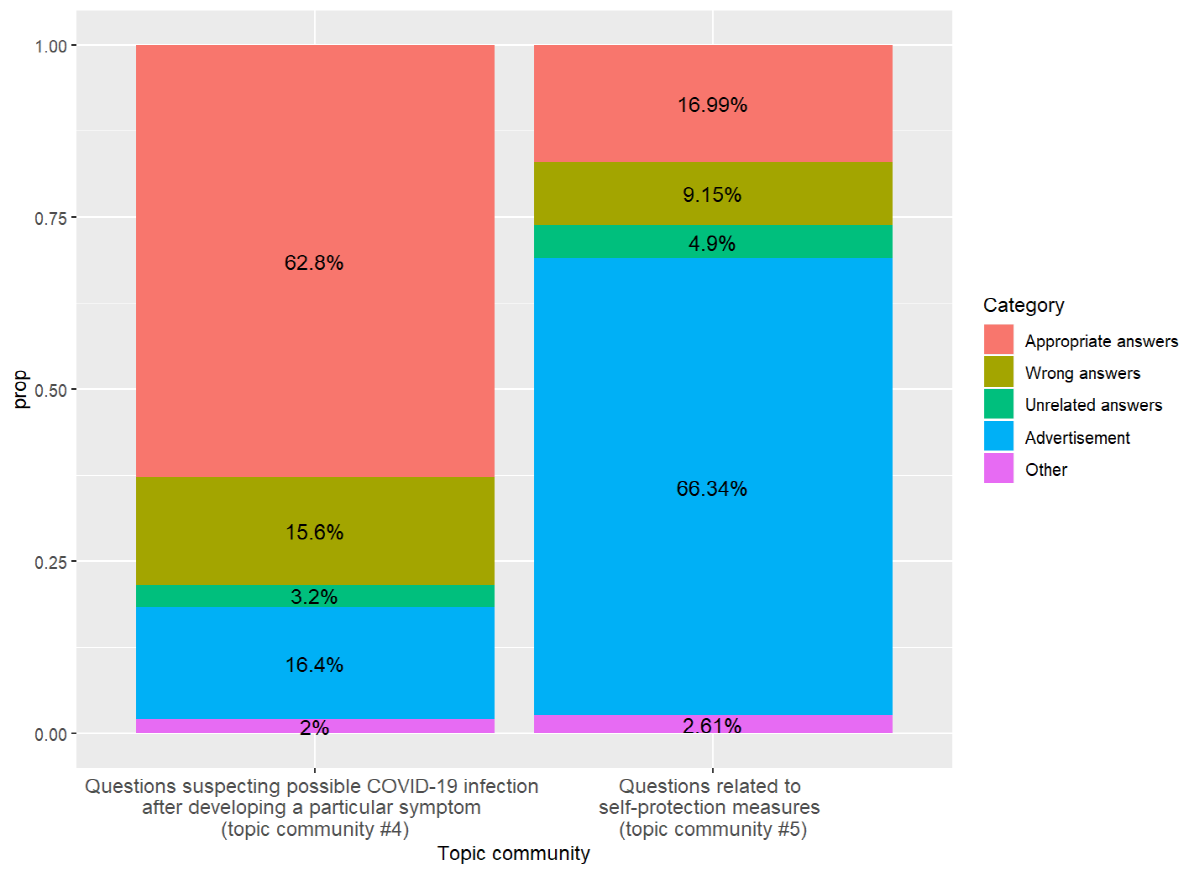
Proportion of answer categories (based on sample data). COVID-19: coronavirus disease; prop: proportion.

Figure 9 shows the results of two medical doctors’ categorization of the answers to sample questions dealing with the main targets of anxiety and worry into five categories.

The answers to questions about physical symptoms were often appropriate and relatively less distorted. On the other hand, there were many advertising answers to questions related to self-protection measures.

## Discussion

In the event of a novel infectious disease outbreak, the general population cannot easily assess the accuracy of the information regarding the disease, and there is increased reliance on online information. Obtaining appropriate and accurate information is extremely difficult, especially in the early stage of an outbreak, due to the uncertainty about the disease. There may be a delay before the governance body, such as health authorities, announces official statements regarding the disease, including symptoms, treatment, or preventive measures. However, it is unlikely that the public would refrain from seeking information and patiently wait until the accurate information becomes publicly available. In this scenario, it is more probable that the members of the populace would seek to acquire the much-needed information from other sources such as the internet.

Consistent with previous studies, our data showed that people sought information regarding COVID-19 on the web. The analysis of 13,136 questions revealed that the largest proportion of topics was regarding anxiety and worries about possible COVID-19 infection after developing a particular symptom. The proportion of topics regarding concerns over working conditions also slightly increased as the COVID-19 outbreak became prolonged. Physical symptoms such as cough, throat pain, and sputum as well as self-protective measures such as wearing a mask were some top key words that simultaneously appeared with the words *anxiety* and *worry* in the word network analysis. This implies that the people were mainly concerned about whether developing a particular physical symptom was relevant to COVID-19 and ways to protect them from COVID-19.

We also analyzed the appropriateness of the answers that replied to the questions. About 63% of the total answers to questions on suspecting possible COVID-19 infection after developing particular symptoms were evaluated as appropriate, while 15.6% of the answers were incorrect, implying a potential dissemination of misinformation. For questions regarding self-protective measures, such as questions asking how to wear masks properly, as much as 66.3% of the answers were advertisements. Thus, it can be assumed that the general population may have difficulty in obtaining appropriate information on self-protective measures.

This study contributes to the establishment of early health communication about public concerns and anxiety observed at the early stage of COVID-19. The initial stage of the epidemic is when neither the health authorities’ policies nor people’s understanding of the epidemic has stabilized. Under these circumstances, people’s online exchange of information and emotion can have a great influence. Governments should implement proper measures to establish online health communication in the early stage of an outbreak to provide appropriate and accurate information. Considering that web data-based studies related to COVID-19 are rare at present, our research’s policy and academic value is more pronounced. Although there are studies that use web data to analyze various characteristics of public psychology for other infectious diseases, there are few studies related to COVID-19. Given that numerous countries have been affected recently and are in the initial stage of the COVID-19 outbreak, our study on South Korea, which experienced COVID-19 relatively early, could be a reference point for policy making in other countries.

This study also contributes to devising methods of measuring public psychology using language data in the circumstance of an infectious disease outbreak. Understanding public psychology and culture is essential when dealing with infectious diseases [28] because public psychology greatly impacts the management of infectious diseases [29]. Public anger toward the infected, for example, is common in infectious disease outbreaks, which can induce the infected to hide from quarantine efforts such as screening tests. This is because the infected people will try to avoid the intense social anger directed at them. To prevent this, it is necessary to promptly measure public sentiment in detail and organize appropriate responses such as creating social support for the infected. However, as previously indicated, traditional surveys are relatively difficult to implement quickly and are not free from limitations (eg, effect of researchers’ frame on answers, relative difficulty of collecting real time data) [30]. Recently, analysis of language data using computers and statistical models has been introduced, and several scholars have suggested its usefulness [13,31,32]. Our paper provides examples of its use concerning an infectious disease outbreak. Beyond the Q&A forum, various language materials are available on the internet, which can be used to actively supplement existing methods of investigation and create diverse methods to approximate public anxiety. In short, this study shows the potential for “online data-based health policy decision making.”

Additionally, this study is differentiated from other studies in how we used text mining techniques. Previously, papers using health-related text data often used frequency as the main information, such as the number of Twitter mentions [33], or applied LDA [34,35], which is the most commonly used topic modeling technique [36]. By using the STM, this study more systematically analyzed how the proportions of COVID-19–related topics varied over time while maintaining the advantages of topic modeling methods. Moreover, this study does not merely apply existing text mining techniques to health-related data but also contributes methodologically on how to use topic modeling methods. Various topic modeling methods have been used as the LDA in many studies [36], most of which draw conclusions from the estimated topics. However, solely focusing on topics has one downside: if the amount of data is substantial, the number of topics will also increase, making it difficult to identify overall patterns that appear in the entire data set. Too many topics, for example 300 topics, represent vast information, which would likely pose a formidable barrier to human researchers. Our research has introduced a way to find sets of topics connected to each other by forming a network of topics and finding cohesive topic communities in them through a network community detection algorithm. In our results, 6 topic communities were found and each community contained content-related topics. It is surprising that the topics belonging to each topic community are meaningfully interrelated, as the topic communities are derived from the community detection algorithm and a correlation matrix deduced by the STM results, not through human researchers’ categorization based on the topics’ contents. In other words, this study proposes a data-driven method of making topic clusters, which could be used for detecting broader themes from numerous topics that were estimated from a vast number of documents.

Various advanced analyses are possible using the data and results of this study. One of the notable results in our study is that the proportion of topic community 4 (questions suspecting possible COVID-19 infection after developing a particular symptom) seems to be linked to the actual number of confirmed patients. Considering that the symptoms observed in topic community 4 (eg, cough, throat pain, and sputum) were reported as common symptoms of COVID-19 in other clinical reports, this linkage might not be a coincidence. This suggests that information extracted from web data may help identify and even predict the actual trend of infectious diseases. Future research could use sophisticated time series analyses to scrutinize this possibility. Additionally, it is noteworthy that a certain kind of question could have a high proportion of answers written from commercial motivations (Figure 9). This implies that there are many attempts to commercially exploit the infectious disease crisis, and the social effects related to these attempts could be explored using this study’s results. Finally, if adequate data of a longer period is available, analyzing how the importance of online information and communication changes over time would be a valuable research project. These further studies will enhance the efficient use of online data for public health.

This study has limitations concerning the range of data. Although Naver is the most popular portal website in Korea, people are not just using this one service. For further comprehensive analysis of information and emotion exchange in online spaces, various sources of web data, including various social network services, need to be incorporated. Furthermore, since the internet user population does not appropriately represent the entire population, it is necessary to consider using the data produced through traditional methods such as surveys and the internet natural language data together.

Nevertheless, this study showed how health information exchanged based on the disease transmission related to people’s anxiety and commercial interest in new infectious disease outbreak via a novel approach using online data analysis and topic modeling.

## Abbreviations

COVID-19: coronavirus disease
FREX: frequency and exclusivity
LDA: latent Dirichlet allocation
Q&A: questions and answers
STM: structural topic model
